# Publisher Correction: Gallium arsenide solar cells grown at rates exceeding 300 µm h^−1^ by hydride vapor phase epitaxy

**DOI:** 10.1038/s41467-019-11927-x

**Published:** 2019-09-03

**Authors:** Wondwosen Metaferia, Kevin L. Schulte, John Simon, Steve Johnston, Aaron J. Ptak

**Affiliations:** National Renewable Energy Laboratory, Golden, CO 80401 USA

**Keywords:** Solar cells, Electronic devices

Correction to: *Nature Communications* 10.1038/s41467-019-11341-3, published online 26 July 2019.

The original PDF version of this Article contained an error in Fig. 3, which was truncated to omit the two right-most data points in each panel. This has been corrected in the PDF version of the Article. The HTML version was correct from the time of publication.

