# Supplementary material for: Gallium arsenide solar cells grown at rates exceeding 300 µm h−1 by hydride vapor phase epitaxy
Source: Nat Commun. 2019 Jul 26;10:3361. doi: 10.1038/s41467-019-11341-3 (PMC6659644; doi:10.1038/s41467-019-11341-3)
Supplement: Supplementary file 1 — Supplementary Information [file 41467_2019_11341_MOESM1_ESM.pdf]

Supplementary information for

Gallium Arsenide Solar Cells Grown at Rates Exceeding 300 μm h^{-1} by Hydride Vapor Phase Epitaxy

Wondwosen Metaferia*, Kevin L. Schulte, John Simon, Steve Johnston, Aaron J. Ptak*

National Renewable Energy Laboratory, Golden, CO 80401 USA

*Corresponding authors

Wondwosen.metaferia@nrel.gov, aaron.ptak@nrel.gov

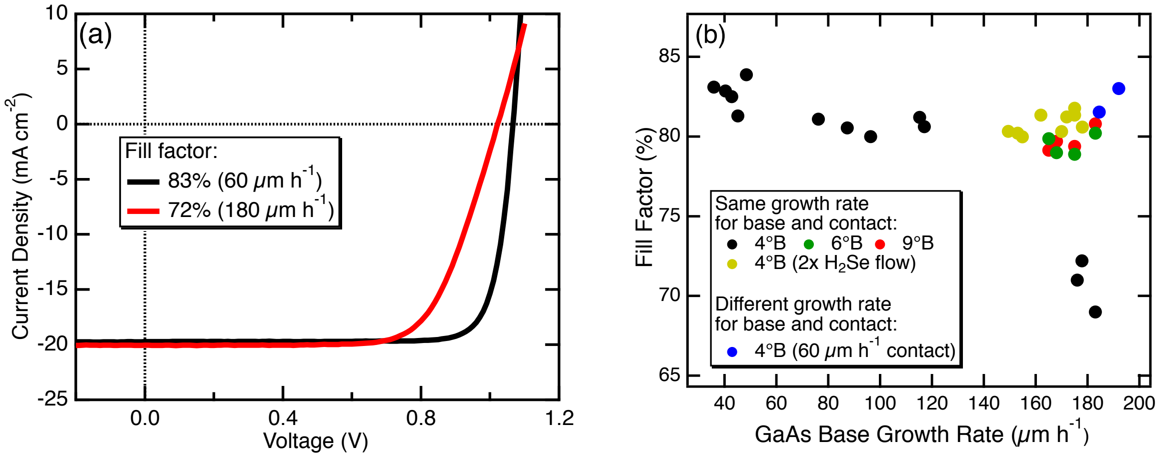

Supplementary Figure 1. Different ways of recovering fill factor for solar cells grown at high growth rates. (a) J-V data for GaAs solar cells grown using the same H_2Se dopant flow and a growth rate of 180 μm h^{-1} in the contact and base layers (red curve) compared with a device grown using a relatively slow growth rate of 60 μm h^{-1} (black curve). (b) Fill factor of GaAs solar cells grown on substrates miscut 4°, 6° and 9° towards (111)B, either with the same growth rate used in the contact and base layers, or a slower contact growth rate (blue circles).

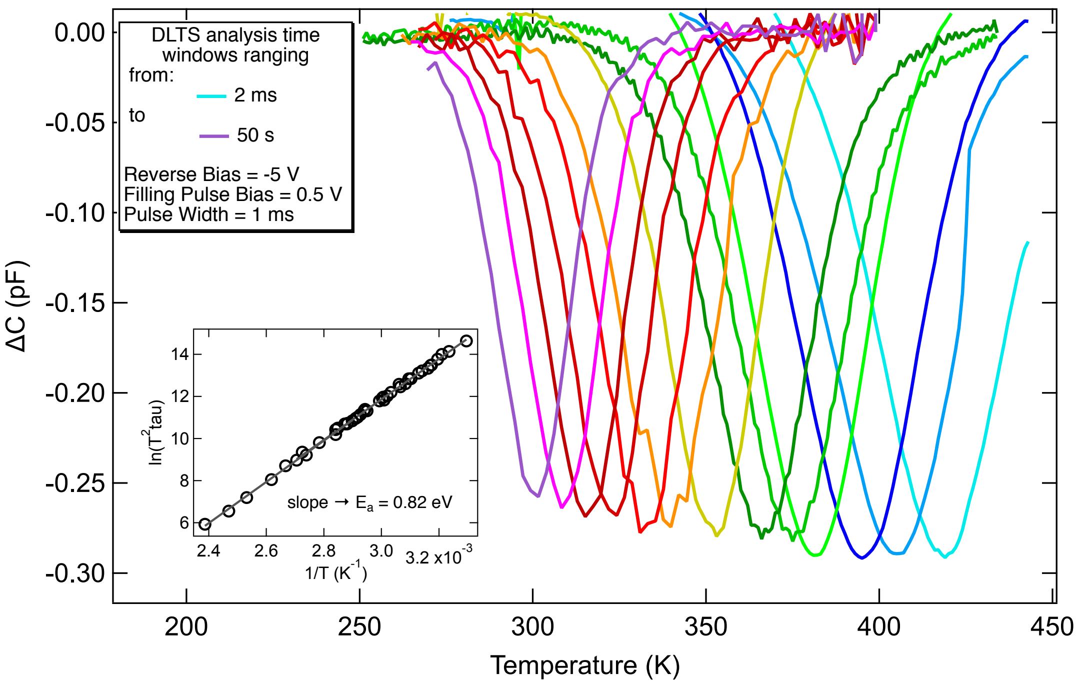

Supplementary Figure 2. DLTS measurement revealed only EL2 type trap in GaAs cells. DLTS rate-window signals as a function of temperature for a sample grown at 180 µm h^{-1}. The inset shows the Arrhenius plot of peak temperatures for corresponding DLTS rate-window time constants.

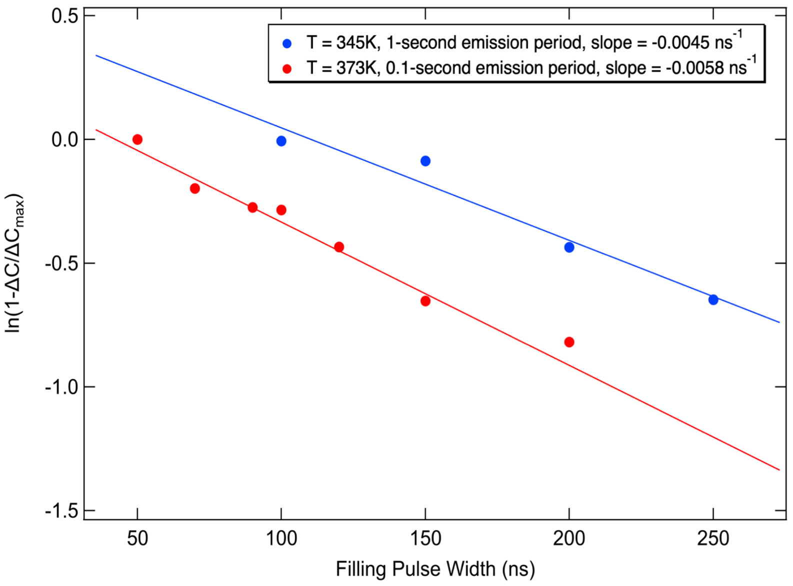

N_D = 2.2 x 10^{16} cm^{-3} (from C-V)
N_t = 3.0 x 10^{14} cm^{-3} (from DLTS)
σ_∞ = 1.3 x 10^{-13} cm^2 (from DLTS)

The slope from the plot is the capture rate, $-c_n$, in ns^{-1}.

$c_n = \sigma v_{th} N_t \qquad v_{th} = \sqrt{\frac{3K_B T}{m^*}}$

where $m^* = 0.067m_o$

For c_n = 4.5 x 10^6 s^{-1} at T=345K,
σ = 3.1 x 10^{-16} cm^2
$\tau = 1/c_n$ = 220 ns

For c_n = 5.8 x 10^6 s^{-1} at T=373K,
σ = 3.8 x 10^{-16} cm^2
$\tau = 1/c_n$ = 170 ns

Supplemental Figure 3. Capacitance transient amplitude at temperatures of 345 and 373 K. Capacitance transient amplitude as a function of pulse width for a sample grown at 180 μm h^{-1}. Calculations are the Shockley-Read-Hall (SRH) recombination lifetime for the device.

Supplementary Note 1. Influence of high growth rate on doping concentration

Recent reports on high-growth-rate MOVPE-grown GaAs solar cells discussed the effect of high growth rate on solar cell design and material quality. [1, 2] For example, Si and Zn dopant incorporation efficiency in the GaAs was shown to increase as the growth rate increased[2] requiring optimization of dopant flows. Supplementary Figure 1(a) compares the *J-V* curves of two D-HVPE-grown GaAs solar cells with GaAs:Se contact layers grown at different growth rates, but with the same H_2Se flows. The fill factor of the device with the high-growth-rate contact layer suffers from increased series resistance compared to the device with the slower contact layer. Transmission line measurements (not shown) indicated that the increased series resistance occurs at the metal/semiconductor interface, and not elsewhere in the device. This effect is likely due to a simple reduction in volumetric dopant density because the same amount of dopant disperses in more matrix material deposited per unit time and represents an issue that must be addressed before high-efficiency devices can be realized.

We grew single-junction GaAs solar cell structures with the same H_2Se dopant flow but different contact layer growth rates to investigate this effect. Supplementary Figure1(b) shows the fill factor measured from the *J-V* curves for samples grown on (100) substrates miscut 4° toward (111)B (black circles) as a function of growth rate. The same growth rate was used for the contact and the base layer in this sample set. This is the simplest arrangement in our D-HVPE reactor because constant reactant flows can be used in the GaAs deposition chamber for the entire device growth. Clearly, the fill factor decreases with contact growth rate, as previously shown in Supplementary Figure 1 (a).

We employed three methods to recover the lost fill factor, focusing on the region of base growth rates around 180 μm h^{-1}. The first and most straightforward path was to increase the H_2Se dopant flow from 6 sccm to 12 sccm (gold circles in Supplementary Figure 1 (b)), which increased the fill factor to higher than 80%, up from about 70%. The second method was to use a hybrid growth rate structure, where base layers were grown at rate of 180 μm h^{-1}, but the contact layer growth rate was slowed to 60 μm h^{-1}. This approach, which requires a change in the reactant flows in the GaAs deposition chamber after deposition of the contact layer before the base is grown, was similarly effective (blue circles in Supplementary Figure 1 (b)). The last approach was to use higher substrate miscuts (6°B and 9°B) than our standard 4°B substrates. Increasing offcut angle towards (111)B was previously shown to enhance incorporation of Se atoms in GaAs.[3] To verify this effect, we grew a set of GaAs epilayers on substrates with varying miscut towards (111)B with a H_2Se flow of 6 sccm and a growth rate of 100 μm h^{-1} (not shown). Carrier concentrations, derived from room temperature Hall effect measurements, increased from 5.0×10^{18} to 1.0×10^{19} to 1.3×10^{19} cm^{-3} when increasing the miscut from 4° to 6° to 9°. We then grew solar cells on substrates with these miscuts using the original 6 sccm H_2Se flow and 180 μm h^{-1} contact layers (green and red circles in Supplementary Figure 1 (b)). The fill factors of the solar cells grown with 6°B and 9°B miscut substrates are ~10 % (absolute) higher than the devices with 4°B miscuts. Our results indicate that all three of the approaches effectively solve the series resistance issue, and we note that the best solution may be application specific. However, increasing the H_2Se flow is likely the simplest solution that maintains the highest overall throughput.

Supplementary Note 2. DLTS capacitance change for various rate-window

Detailed analysis is illustrated for the sample grown at 180 μm h^{-1} on a 4°B substrate. DLTS-based changes in capacitance for various rate-window time constants are plotted in Supplementary Figure 2. A negative peak corresponds to trapping and emission of majority-carrier electrons, and there is only one visible in the collected data. The values for each analyzed time constant (τ) are plotted against their corresponding peak temperatures in an Arrhenius plot as shown in the inset of Supplementary Figure 2. The slope of the linear fit corresponds to an activation energy of 0.82 eV, consistent with an EL2 defect in GaAs.

Supplementary Note 3. DLTS capacitance transient as a function of pulse width

As shown in Supplementary Figure 3, we measured the magnitude of the capacitance transient as a function of the filling-time pulse-width in the range of 50 to 250 ns to directly measure the trap's electron capture rate. This slope value is used to calculate an electron capture-cross-section of 3 to 4×10^{-16} cm^2. With the measured EL2 defect density of 3.0×10^{14} cm^{-3} and measured capture cross section at 345 K of 3.1×10^{-16} cm^2, the defect-related lifetime is 220 ns, which is longer than the 125 ns radiative recombination lifetime of the n-GaAs base layer in our devices.

Supplementary Reference

(1) Schmieder, K. J.; Armour, E. A.; Lumb, M. P.; Yakes, M. K.; Pulwin, Z.; Frantz, J.; Walters, R. J. Effect of Growth Temperature on GaAs Solar Cells at High MOCVD Growth Rates. *IEEE J. Photovolt.* **2017**, *7* (1), 340–346.

(2) Schmieder, K. J.; Yakes, M. K.; Bailey, C. G.; Pulwin, Z.; Lumb, M. P.; Hirst, L. C.; González, M.; Hubbard, S. M.; Ebert, C.; Walters, R. J. Analysis of GaAs Solar Cells at

High MOCVD Growth Rates. In *2014 IEEE 40th Photovoltaic Specialist Conference (PVSC)*; 2014; pp 2130–2133.

(3) Sun, Y. T.; Anand, S.; Lourdudoss, S. Crystallographic Orientation Dependence of Impurity Incorporation during Epitaxial Lateral Overgrowth of InP. *J. Cryst. Growth* **2002**, *237–239*, 1418–1422.

(4) Kondo, M.; Anayama, C.; Okada, N.; Sekiguchi, H.; Domen, K.; Tanahashi, T. Crystallographic Orientation Dependence of Impurity Incorporation into III-V Compound Semiconductors Grown by Metalorganic Vapor Phase Epitaxy. *J. Appl. Phys.* **1994**, *76* (2), 914–927.
